# Riding the Wave: Reconciling the Roles of Disease and Climate Change in Amphibian Declines

**DOI:** 10.1371/journal.pbio.0060072

**Published:** 2008-03-25

**Authors:** Karen R Lips, Jay Diffendorfer, Joseph R Mendelson, Michael W Sears

**Affiliations:** 1 Department of Zoology, Southern Illinois University, Carbondale, Illinois, United States of America; 2 Illinois Natural History Survey, Champaign, Illinois, United States of America; 3 Zoo Atlanta, Atlanta, Georgia, United States of America; Imperial College London, United Kingdom

## Abstract

We review the evidence for the role of climate change in triggering disease outbreaks of chytridiomycosis, an emerging infectious disease of amphibians. Both climatic anomalies and disease-related extirpations are recent phenomena, and effects of both are especially noticeable at high elevations in tropical areas, making it difficult to determine whether they are operating separately or synergistically. We compiled reports of amphibian declines from Lower Central America and Andean South America to create maps and statistical models to test our hypothesis of spatiotemporal spread of the pathogen Batrachochytrium dendrobatidis (*Bd*), and to update the elevational patterns of decline in frogs belonging to the genus *Atelopus*. We evaluated claims of climate change influencing the spread of *Bd* by including error into estimates of the relationship between air temperature and last year observed. Available data support the hypothesis of multiple introductions of this invasive pathogen into South America and subsequent spread along the primary Andean cordilleras. Additional analyses found no evidence to support the hypothesis that climate change has been driving outbreaks of amphibian chytridiomycosis, as has been posited in the climate-linked epidemic hypothesis. Future studies should increase retrospective surveys of museum specimens from throughout the Andes and should study the landscape genetics of *Bd* to map fine-scale patterns of geographic spread to identify transmission routes and processes.

## Author Summary

Once introduced, diseases may spread quickly through new areas, infecting naive host populations, such as has been documented in Ebola virus in African primates or rabies in North American mammals. What drives the spread of the pathogenic fungus Batrachochytrium dendrobatidis (*Bd*), which causes chytridiomycosis, is of particular concern because it has contributed to the global decline of amphibians. We modeled the spatiotemporal pattern of the loss of upland amphibian populations in Central and South America as a proxy for the arrival of *Bd* and found that amphibian declines in Central and South America are best explained by *Bd* spreading through upland populations; we identified four separate introductions of *Bd* into South America. Climate change seriously threatens biodiversity and influences endemic host–pathogen systems, but we found no evidence that climate change has been driving outbreaks of chytridiomycosis, as has been posited in the climate-linked epidemic hypothesis. Our findings further strengthen the spreading-pathogen hypothesis proposed for Central America, and identify new evidence for similar patterns of decline in South American amphibians. Our results will inform management and research efforts related to *Bd* and other invasive species, as effective conservation actions depend on correctly identifying essential threats to biodiversity, and possible synergistic interactions.

## Abstract 3

The spread of chytrid fungus, rather than climate change, best explains amphibian declines in Central and South America, based on an analysis of observed epidemics with predictable spatiotemporal patterns in four of five mountain ranges.

## Introduction

Amphibian populations are declining across the globe at an alarming rate, with over 43% of species in a state of decline [1,2]. In addition to long-recognized threats such as habitat loss, overexploitation, and exotic species introductions, amphibians in all biogeographic regions face several new threats, including climate change, emerging infectious diseases, and chemical contaminants [3]. Since amphibian declines were first noticed in the late 1980s [4], many studies have attributed certain declines to a particular cause, often through a process of elimination and using limited data [5], rather than through a rigorous testing of hypotheses [6,7]. This type of approach is particularly a problem for remote tropical areas for which even baseline biotic inventories are scarce or nonexistent, and for countries with limited funding or scientific infrastructure [8]. Yet, these areas harbor the greatest number of amphibian species and are suffering the greatest numbers of declines and extinctions [2]. From a scientific standpoint, it is important to evaluate and test likely hypotheses of declines, inform conservationists with data-based predictive scenarios, and design research to provide additional data from new and understudied sites and taxa. From a practical standpoint, we need solid data and strong hypotheses to better plan conservation activities [9,10]. From an ethical standpoint, we need to understand, as quickly as possible, the global patterns and causes of amphibian declines to prevent further losses of biodiversity. In this spirit, we examine alternative hypotheses regarding the relationship between climate change and amphibian declines, including extinctions.

One cause of amphibian declines is chytridiomycosis, hypothesized to be an invasive disease [11] recently introduced into the Americas and Australia, that is caused by the fungal pathogen Batrachochytrium dendrobatidis (“*Bd*”). *Bd* is widespread throughout South America [12–16], and its role in population declines has been linked to interactions with climate change, although no studies have explicitly considered evidence of spatiotemporal spread of disease as an alternative to the recently proposed climate-linked epidemic hypothesis (CLEH) [17,18]. Regardless, both field studies on amphibians [6,19] and on fungal population genetics [20–21] strongly suggest that *Bd* is a newly introduced invasive pathogen. These case studies, from Central America and western North America [6,22], describe healthy amphibian populations when *Bd* is absent, but show acute die-offs and subsequent population declines immediately following detection of *Bd* at study locations. Results in both regions parallel other well-studied disease systems (e.g., mammalian rabies, Lyme disease, and Ebola virus [23–27]), where invasive diseases spread across the landscape, invading new areas and affecting naive populations. Such results (although not framed as rigorous tests of hypotheses) suggest an outcome for future declines with some degree of certainty [23,28].

Predicting the impacts of an invasive disease will require an understanding of the biotic and abiotic factors that influence the interactions between the host and pathogen. Temperature and moisture influence most aspects of the biology of amphibians [29,30]. Thus, climate change likely will impact amphibians as a result of both direct effects on physiology and as a result of indirect effects following changes in interactions with other species. Indeed, responses to climate change by amphibians are numerous (reviewed by [31]) and may occur on the scale of individuals [32], populations [33–35], and species [13]. Documented effects of climate change on amphibians typically have been in the form of population fluctuations or long-term declines, rather than sudden die-offs and subsequent rapid declines in local populations.

Just as temperature, rainfall, and humidity influence the biology of amphibians, so too might these factors affect the growth, persistence, and ecology of a potential pathogen. Changes in regional or local climate may directly or indirectly alter pathogen development and survival rates, disease transmission, and host susceptibility [36], thereby influencing the degree of host population response. Like amphibians, much of the basic biology of *Bd* is affected by temperature and moisture. In the lab, optimal growth occurs between 17–25 °C, and death occurs at temperatures above 29 °C or below 0 °C, or after prolonged desiccation [37]. Such lab results are reflected in field studies from Australia, Panama, South Africa, and the western US (e.g., California and Colorado) in which prevalence of infection varies by season, elevation, or region [38], with increased prevalence associated with cooler temperatures and moister conditions [39–43]. All of these studies occurred at locations where *Bd* had been introduced prior to the study, and was endemic during the course of research. Although environmental factors likely influence the survival and growth of *Bd*, there is no evidence that climatic factors cause outbreaks of chytridiomycosis from resistant spores or from saprophyte forms. The lack of evidence for climate-induced outbreaks is especially important to note, given recent suggestions that promote climate change as a potential mechanism for amphibian declines [17].

Recent global climate change is well documented [44] and is confounded in time with the recent declines of amphibians [2], which requires careful analyses to distinguish correlation from causation. In Central America, temperature is predicted to increase, and rainfall is predicted to decrease [44], making many of these areas less favorable for *Bd*. Paradoxically, the CLEH predicts that amphibians should decline in years that follow an unusually warm year because “shifts in temperature influence disease dynamics” ([17] p. 161). Under this scenario, *Bd* is proposed to emerge from a dormant state, or switch from a facultative saprobe into a pathogen as the environment becomes warmer. Specifically, the CLEH proposes that outbreaks of chytridiomycosis are triggered by a shrinking thermal envelope, in which maximum temperatures become cooler, and minimum temperatures become warmer [17]; this effect is hypothesized to be most pronounced at mid-elevations. The CLEH was based on relating timing in changes of regional temperature to the timing of the disappearance of species of harlequin frogs (genus *Atelopus*) in Lower Central and northern South America [14]. Pounds et al. [17] presented analyses to assess associations of pan-Neotropical weather patterns and the putative timing of the disappearance of *Atelopus* species [14]. Oddly, the authors did not examine the database for evidence of simple spatiotemporal patterns in the disappearance of *Atelopus* species, which might be suggestive of epidemic movement of disease.

In this paper, we evaluate the data regarding the declines of *Atelopus* species [14], as well as additional data from other amphibian species in Lower Central America and Andean South America derived from published literature and from our own field work. We search for spatial and temporal patterns of amphibian declines to determine whether evidence supports an alternative hypothesis that *Bd* is an invasive pathogen. If *Bd* represents a disease epidemic, then spatiotemporal patterns would indicate that after an initial introduction, the pathogen spreads systematically through environments with favorable climates, geography, and host populations. Further, if *Bd* is an exotic invasive [6] in South America, then spatiotemporal patterns should be similar to those documented in Central America and Australia [6,45].

## Methods

### Amphibian Decline Data

Because few die-offs and population declines of amphibians have ever been observed directly, especially prior to the 1990s, attribution of the causes and the timing of these events is often only a rough estimate, and in many cases, no additional data will ever be available. Furthermore, in the case of *Atelopus*, taxonomic studies are still discovering “new species” decades after the organisms have gone extinct [46]. The conservation status of this group was summarized [14] against a backdrop of taxonomic confusion, widespread habitat destruction, remote localities, sociopolitical challenges, and in the absence of population data for most *Atelopus* species. These uncertainties led to the conclusion that “in many cases the available information did not permit quantitative analysis.” ([14] p.192).

Findings [14] were presented in the form of “Last Record” to indicate the last known sighting of a live individual in the field, and were often based on the last known museum specimens collected. The conservation status of each species was classified as “Stable,” “Decline,” or “Data Deficient,” and well-qualified causes (e.g., habitat loss, introduced predatory fish, *Bd*, and other, unknown factors) of the apparent declines were presented [14]. Many of the detailed notes and comments regarding the suspected causes of declines did not appear in the final publication, but a final draft of the full database (dated 15 March 2004) was circulated among the coauthors and to other members of the RANA network (http://rana.biologia.ucr.ac.cr/). These data were used [17] to test the proposed CLEH. They renamed “Last Record” as “Last Year Observed” (LYO), and used those dates as a proxy for the actual date of disappearance of individual species. Realizing that the data contained uncertainties, the following justification was presented:

“Undoubtedly, the LYO does not accurately represent the timing of a disappearance in some cases, especially in tier two. Thus uncertainty is high for any particular species, and the strength of our conclusions lies in the broad patterns. Errors in the data could generate these patterns [of correlation of timing of disappearance with unusually warm years] only if sampling were biased so that the LYO tends to follow a relatively warm year irrespective of the timing of disappearances. A decline in a cool year might be misclassified as having occurred in a warm year, *but the reverse is no less probable*.” (see Supporting Online Material p. 18 in [17]) (emphasis ours).

### Date of Decline versus Last Year Observed

**Figure 1:**
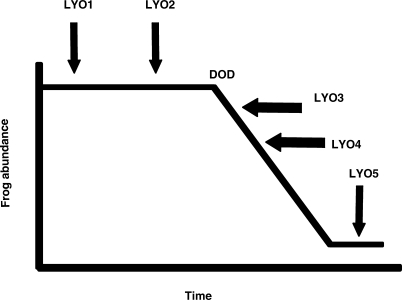
Graphical Representation of the Various Relationships Possible between DOD and LYO

Ultimately, testing hypotheses such as the CLEH or *Bd* as an invasive pathogen requires data about the spatiotemporal patterns of *Bd* occurrence. Unfortunately, little sampling of *Bd* from nondeclining populations or from the environment (independent of frogs) has been undertaken, with the result that frog decline has been used as a proxy for *Bd* appearance. This leads to an important distinction between LYO data [17] and the actual time when *Bd* arrived at a location. When using amphibian population declines to study spatiotemporal patterns of *Bd*, the datum of interest is the date of the actual decline (DOD, hereafter) because it more accurately reflects the timing of the arrival of *Bd* to a site than does LYO (Figure 1).

LYO, as the year of last record [14], may not reflect *Bd* dynamics for a number of reasons. First, because the entire range of *Atelopus* species could not possibly be surveyed in a systematic fashion, LYO might indicate the last time scientists visited a particular site and noted a particular species [14]. Second, species occurring in remote or politically unstable areas have not been surveyed in decades [14]. Third, rediscovery of a few individuals changes LYO, despite a known DOD. For example, A. mucubajiensis declined between 1988 and 1990 [47–49], but a rediscovery of a few individuals in 2004 [17] produces an LYO 14–16 y after the DOD. A similar example is the case of A. bomolochos in Ecuador. The species declined in 1980 [50], but has an LYO of 2002, based on a singular sighting decades after its DOD, as noted in the original 15 March 2004 database: “One individual seen in 2002 in the PN Sangay (D. Almeida), but otherwise this formerly abundant species has disappeared from its range.” This species had an initial status of Decline [14], and infected individuals were found in 1980 and 1991. It was later reclassified as Stable [17], although data indicate this species declined precipitously in the 1980s (likely due to *Bd*), with a singular sighting in 2002.

### Effects of Error in the Analyses

Pounds et al. [17] acknowledged the *Atelopus* dataset contained uncertainties and errors (see above). However, their analytical approach did not explicitly test how error around LYO—at any quantified level—affected the regression analyses that were performed to assess relationships between temperature and timing of disappearance. The bootstrapping techniques used by [17] calculate confidence intervals for estimated parameter values from a given set of data [51]. Though used on a wide array of subsets from the original data (e.g., separate tiers of frogs, rediscovered species, and species in protected areas), this approach did not explicitly address the potential effects of the acknowledged errors in LYO on the estimated correlation coefficients.

Elementary statistical theory indicates that increases in sampling error, independent of bias in the parameter estimator, will reduce model fit simply by increasing the variance in the variables. In the dataset, DOD differed from LYO for 24–27 of 54 species, ten of these were Stable despite a reported LYO, and for those species with different dates for LYO and DOD, the mean difference was 11.2 y ± 8.2 (standard deviation [SD]) (Table 1). Thus, previous analyses [17] using LYO data had large amounts of sampling error relative to the 1-y time lag used in the regression models [14,17]. Given the low accuracy of the data to estimate *Bd* in time, we wanted to determine whether the data could support such fine resolution of statistical relationships between species declines and temperature. As such, we performed an explicit examination of how the observed sampling error in LYO would affect correlations between population declines and air temperature (AT) [17]. We perform similar simulations on our new analyses correlating time since probable infection and distance of spread.

**Table 1:**
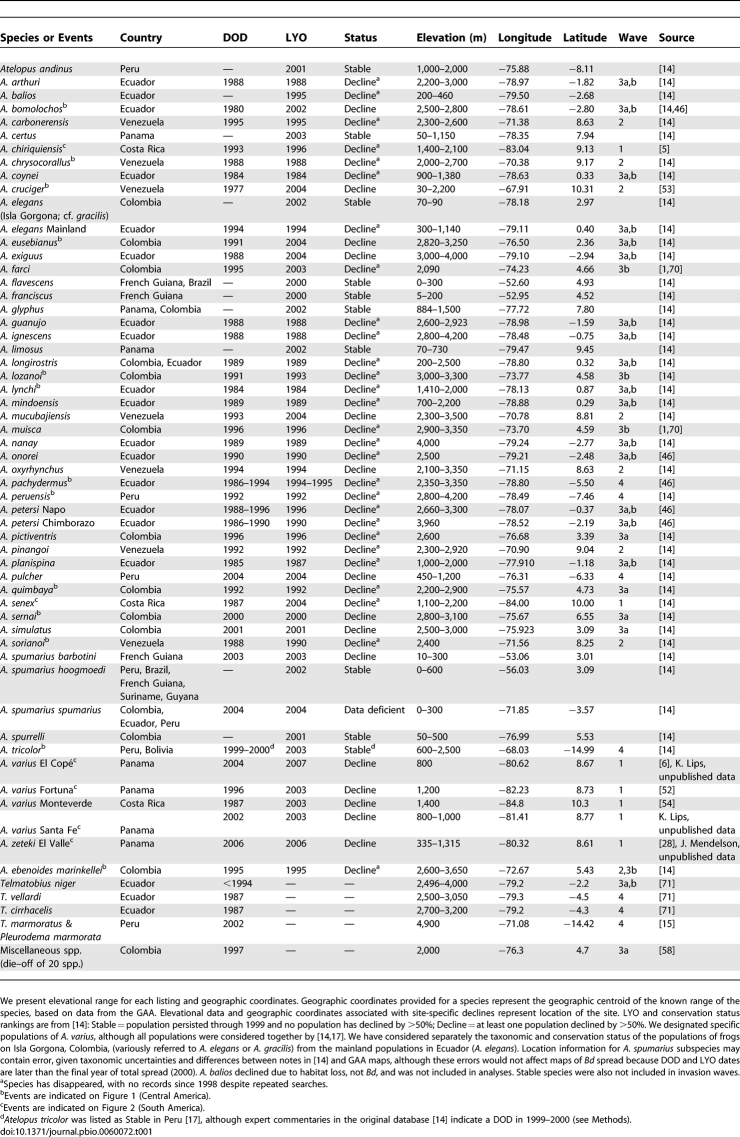
List of Species and Approximate Date of Decline (DOD) Included in Our Analyses

**Figure 2:**
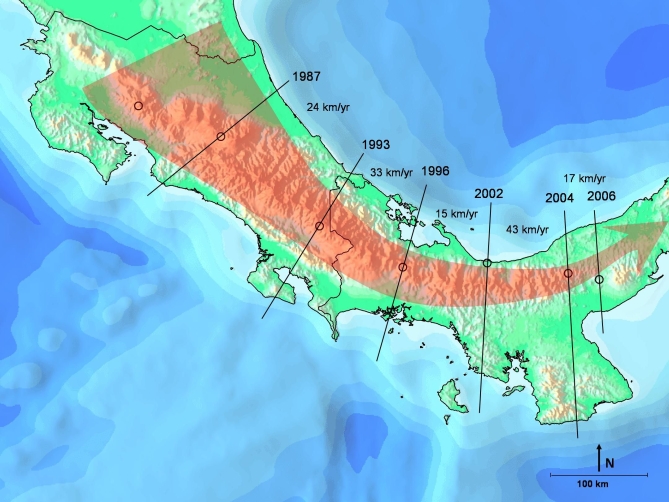
Map of Central American Spreading Wave (Wave 1) with DOD Sites Indicated and Rate of Spread DOD sites are indicated by open circles. Black bars indicate the hypothesized leading edge of the wave of *Bd* in the year indicated*.*

We tested the robustness of the relationship between AT and LYO data reported in [17] (see Figure 3 in [17]), using a Monte Carlo analysis to examine the effect of systematic error on LYO date that might be expected from sampling error for Tier 1 species. First, we added bidirectional error around LYO data sampled from a normal distribution, with a mean of zero and an increasing standard deviation of up to 6 y (a conservative amount given the observed differences). This type of error would imply that either a DOD (not necessarily an extinction event) occurred sometime around the LYO date, or that because searches for individuals used in LYO dates were not systematic, the true LYO date could have been missed. Next, we added error forward in time only, indicating that the real date of extinction occurred sometime after the last observed individual was noticed by observers in the field. This error could especially be likely given that individuals from populations at low density might be difficult to detect and because, as above, searches for individuals were not systematic. This directional error was modeled in three ways. First, we added error to LYO sampled from a uniform distribution, with error ranging from 0 to 6 y (i.e., LYO was as likely to occur some time after the reported date as it was likely to occur at some time close to the reported date). Second, we added error sampled from a Poisson distribution, with a mean error from 0 to 4 y (i.e., the actual extinction date likely occurred closer to the reported LYO date than later). Third, we similarly added error sampled from an exponential distribution, with a mean error from 0 to 6 y. For the Monte Carlo simulations, we ran 10,000 trials for each year of error that was added to LYO, for all types of error additions. For each trial, we calculated the correlation coefficient of the relationship between the resultant LYO and AT following the time interval 1970–1998 [17]. For each randomized trial, we report the mean correlation coefficient and a 95% confidence interval. Further, we report the number of trials out of 10,000 that produced a result equal to or greater than the magnitude of the result reported in [17]. A low number of such trials would suggest that the result from the original analysis is not a reliable estimate for the true relationship between climate and LYO.

**Figure 3:**
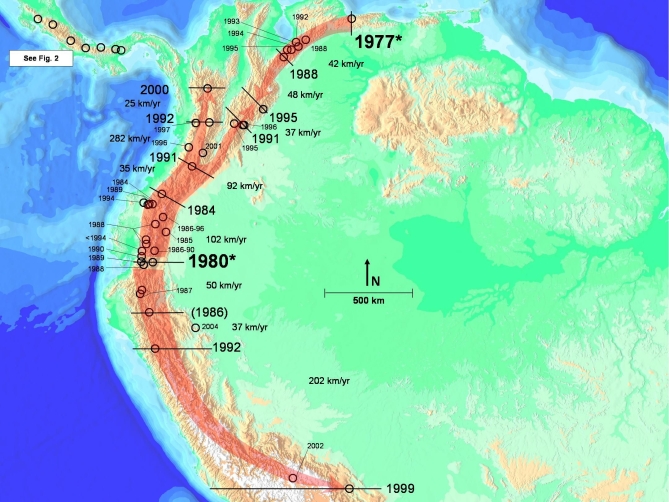
Map of South American Waves (Two Introductions; Waves 2, 3a, 3b, 4; See Text for Description of Waves), with DOD Sites Labeled and Rates of Spread Indicated between Black Bars (1986) indicates A. pachydermus had an estimated DOD from 1986–1994, and we used the 1986 date. If we had used the 1994 date, the demarcation for spread would have moved north to the nearby 1987 locations. Earliest dates are in larger font and marked with an asterisk.

### Estimation of Dates of Declines

We gathered records of approximate DOD and *Bd* infection (Table 1) from published studies based in South and Central America, from the Global Amphibian Assessment (GAA) [1], from the *Atelopus* database [14], and from our own field work. In the case of the *Atelopus* database [14], we excluded species that persisted at typical levels of abundance (Stable [14]), species that were Data Deficient, and lowland species for which evidence of disease- or climate-induced declines was lacking. In these analyses, we used species (or groups of species at a site) for which there was a reasonable estimate of the year of actual decline. For purposes of comparison, we present (Table 1) both our estimate of DOD and the estimate of LYO ([14,17] and other sources cited in Table1). Geographic coordinates for each species (Table 1) represent the geographic centroid of the known range of the species derived from the GAA database (V. Katariya, personal communication). Elevational ranges were taken from the GAA. In the case of site-specific declines, elevational data and geographic coordinates represent the data for that site.

Rates (kilometers/year) of spread were calculated by dividing the distance between pairs of locations by the interval between the DODs. Rates of spread in Central America (our Wave 1) have been described previously [5,6]. These data were updated and recalculated by supplementing the original database with DOD data with additional published records (Table 1; Wave 1).

### Estimation of Spatiotemporal Patterns of Disease Epidemics

We propose *Bd* as an exotic introduction with subsequent spread as an alternative hypothesis to the CLEH. We created a map of known locations of *Bd* by date using DOD and geographical data (Table 1). Starting with the earliest record, we built temporal demarcations of *Bd* movement that depicted the fastest observed spread by finding locations that were both the farthest away and with the shortest time interval from the previous earlier DOD. We created maps along each of five primary cordilleras assuming *Bd* could not move through lowland areas, given its thermal requirements. By creating demarcations using consecutive years of data, we could determine whether the area covered by *Bd* increased in size through time, as expected if *Bd* were to spread via a wave-like expansion. Our null hypothesis was that little or no wave-like pattern would indicate that *Bd* presence was randomly scattered across space and time (e.g., [27]).

Further, for each wave, we ran additional regression analyses [27] to quantitatively test for a linear relationship between time and space in *Bd* spread. Distances between the origin of disease introduction and DOD location were regressed against the differences in time between the earliest DOD and the date of introduction within a particular wave. We did not account for autocorrelation among geographic locations in our analysis because spatial relationships were modeled directly as the causal factor underlying the temporal patterns of disease. Such a correction would have been necessary if we had sought to explain temporal patterns of disease with some factor other than spatial distance (e.g., altitude, climate, and habitat type). Significance for the regression was determined through randomization techniques [52] to ensure robustness against any violations of normality in the data [52]. Ten thousand random datasets were constructed by pairing values of distance with values of time that were drawn randomly (with replacement) from the pooled distribution of times in the dataset within a wave. Regressions were then calculated for each of these random datasets. The significance of the original regression was determined by comparing the β from the original regression with those from regressions of the randomly constructed datasets. The significance level of the test was calculated as the proportion of values in the distribution of β's from the randomized datasets that were as extreme as or more extreme than the β calculated in the original regression. A significant (*p* < 0.05) regression coefficient supports disease spread through space and time whereas a nonsignificant regression indicates random occurrences of disease across space and time. This approach allows for testing the competing hypotheses that *Bd* is either a spreading, invasive pathogen or an emerging, endemic pathogen, and is similar to issues and statistical methods regarding Ebola outbreaks in African primates and humans [27].

Similar to our approach for LYO, we developed a Monte Carlo procedure that simulated error in DOD to determine how robust the regression analyses of each wave were to sampling error in DOD. For each wave, we added error sampled from a uniform distribution to each datum backward in time for up to 20 y. For the error simulations on each wave, we ran 10,000 trials for each year of error that was added to the time since earliest DOD. From these trials, we calculated the mean and 95% confidence interval on the slope of the relationship (i.e., β) between the number of years since the earliest DOD against distance from the earliest DOD using linear regressions. We considered the amount of error necessary to falsify the hypothesis of an epidemic wave as that when the 95% confidence interval of the slope overlapped zero.

The earliest records of *Bd* in South America are from A. bomolochos in Cañar Province, Ecuador, in 1980 [12]; another relatively old record from 1986 (associated with a DOD ca. 1977; Table 1) exists from A. cruciger, near Caracas, Venezuela [53]. Consequently, we used these as two starting points, with the first assuming a *Bd* introduction in Cañar, Ecuador with subsequent spread, and the second assuming an introduction near Caracas, Venezuela (Wave 2). We calculated the rates of spread from Cañar (1) northward into Colombia along the Cordilleras Occidental, Central, and Oriental (Waves 3a); (2) northeastward into Venezuela along the Cordillera Oriental of Ecuador and Colombia and into Los Andes de Merida (Wave 3b); and (3) southward into Peru along the Cordillera Occidental and Oriental (Wave 4).

We calculated rates of spread assuming the Venezuela record represents a second, independent introduction of *Bd* into Andean South America (Wave 2). If this is the case, the Venezuelan introduction likely represents the third introduction onto the continent, as the second oldest record of *Bd* in South America is from the Atlantic coastal forest of Brazil in 1981 [16]. This study focuses on the Lower Central America and Andean Region of South America, so we do not include these Atlantic Brazilian records in the calculations here; evidence of an epidemic wave in Brazil was discussed previously [5].

### Elevation and Declines in Atelopus Species

According to the CLEH, extinction risk for *Atelopus* species is greatest at mid-elevations, between 1,001 and 2,399 m [17] (see Figure 1 in [17]). Using the same dataset, LaMarca et al. [14] originally concluded that no species of *Atelopus* above 1,500 m was Stable. The differences in conclusions between these two analyses were striking and were likely caused by two factors. First, the use of LYO [17] excluded information about status [14]. Second, while using LYO data as a proxy for the percentage of species missing by elevation, Pounds et al. [17] used a cut-off of 1998 to determine status from the LYO data. Species observed between 1999 and 2004 (final year in the database) were categorized as having neither declined in abundance nor gone missing. The justification for 1998 as a cut-off date was apparently that no LYO of 1999 was recorded, thus representing “a natural break in the data” (see Supporting Online Material, p. 24, of [17]).

The use of LYO instead of DOD, in combination with the arbitrary 1998 cut-off date [17], could have obscured possible elevational patterns of decline in *Atelopus*, so we analyzed Status data [14] with no temporal cutoffs to determine elevational patterns in declines. We used the same elevation categories as [17] and included only species categorized as Stable or Decline, excluding species considered Data Deficient. We considered A. tricolor as Decline (contra [17]) based on expert commentaries in the original *Atelopus* database [14] (see comments above). We calculated an exact Pearson chi-square test statistic for the four elevation classes in a two-way (Stable or Decline) contingency table because some cells had zero species.

### Chytridiomycosis and the Decline of Amphibians at Monteverde, Costa Rica, and in Ecuador

Although it is widely assumed that the decline of amphibians in 1987 at Monteverde Cloud Forest Reserve, Costa Rica, was the result of an outbreak of *Bd*, direct evidence of such does not exist. A positive record of *Bd* in 2003 (Supporting Online Material, p. 3, of [17]) indicates that *Bd* is now endemic to the area. Because the CLEH was based on patterns of climate change and the loss of A. varius at Monteverde [54,55], we examined museum specimens for evidence of presence of *Bd* prior to 1986. We collected skin samples from the pelvic patch of 64 frogs collected between 1979 and 1984 from mid-elevations (985–1,645 m). The specimens represented 18 species of anurans, 12 of which are riparian and 11 of which have been shown to be infected by *Bd* elsewhere in their range (Table S1). Skin samples were stored in 70% alcohol, transported to the histology lab at Southern Illinois University, Carbondale, where we followed histological procedures as described in [56]. We calculated *Bd* infection prevalence and 95% Clopper-Pearson binomial confidence intervals for the 64 samples.

Merino-Viteri [57] conducted a similar histological survey of 174 museum specimens representing more than 16 species, collected between 1953 and 2000, from throughout Ecuador. We calculated *Bd* infection prevalence and 95% Clopper-Pearson binomial confidence intervals to determine the likelihood of detection if *Bd* were present prior to evidence of die-offs. For Ecuador, we used two subsamples of this database [57]: (1) 32 specimens (29 A. ignescens and three other Atelopus spp.) collected prior to 1980, and (2) 89 specimens of Atelopus ignescens sensu strictu [46] collected between March 1953 and November 1987.

## Results

### Directional Spread

We found evidence of directional spread of *Bd* along most of the principal cordilleras of Lower Central America and the Andean region (Figures 2 and 3), supporting the hypothesis that *Bd* is an exotic pathogen that was introduced into South America in the late 1970s–early 1980s, and has caused multiple amphibian declines in the past 30 y [6,12–14,58]. The maps indicate localized areas of initial introductions and subsequent expansion of moving wave fronts.

Although the oldest record of *Bd* is in the environs of Cañar, Ecuador, we find questionable the hypothesis that this record represents a singular introduction of the pathogen to all of South America. If this were the case, then the subsequent northward and eastward spread toward Caracas, Venezuela, in a mere 5 y left no trace of itself in the intervening regions (e.g., Colombia). This rate of movement (∼300 km/y; Table 1) would be much higher than any of the observed rates of spread for the region using more accurately defined intervals (Table 1). Instead, we prefer the more parsimonious hypothesis of dual introductions near Cañar, Ecuador [12], and near Caracas, Venezuela (Figure 3). The hypothesized introduction into Venezuela is based on the earliest record there of *Bd* (A. cruciger, 1986), at a site located 115 linear kilometers from Caracas, and reports of population declines in that species in the mid-1970s [53]. Given the proximity of the geographical centroid of A. cruciger, and the potential error in DOD, the data support the hypothesis of a second Andean introduction near Caracas in the mid-1970s, and subsequent westward spread along the Merida Andes–Cordillera Oriental axis towards Colombia and Ecuador. Our analyses indicate that *Bd* invaded the Cordilleras Occidental and Central of Colombia, moving northward towards Panama, and moved northeast towards Venezuela along the Cordillera Oriental–Los Andes de Merida axis. Precisely where the eastward expansion from Ecuador and the westward expansion from Caracas meet—likely in Colombia—cannot be determined accurately given the available data.

**Figure 4:**
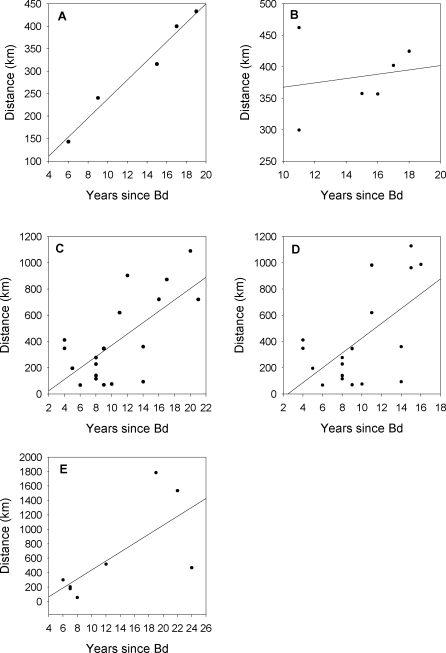
Evidence for Spatiotemporal Spread of Chytridiomycosis in Five Waves in Central and South America The relationship between time since the earliest DOD within a wave and distance of spread of *Bd* was significant for (A) Wave 1 (*p* = 0.0067; β = 21.17; *R* ^2^ = 0.97), (C) Wave 3a (*p* = 0.00153; β = 43.32; *R* ^2^ = 0.47), (D) Wave 3b (*p* = 0.0050; β = 56.57; *R* ^2^ = 0.40), and for (E) Wave 4 (*p* = 0.0279; β = 61.95; *R* ^2^ = 0.49), but the relationship for (B) Wave 2 was not supported (*p* = 0.32).

Overall, there is support for directional spread of *Bd* (Figure 4). The relationship between time since the earliest DOD within a wave and distance of spread was significant for Wave 1 (*p* = 0.0067; β = 21.17; *R* ^2^ = 0.97), Wave 3a (*p* = 0.0013; β = 43.32; *R* ^2^ = 0.47), Wave 3b (*p* = 0.0050; β = 56.57; *R* ^2^ = 0.40), and Wave 4 (*p* = 0.0279; β = 61.95; *R* ^2^ = 0.49). The relationship for Wave 2 was not supported (*p* = 0.32). Note that Wave 1, sampled with nearly no error in DOD, had much higher *R* ^2^ values than the other waves, suggesting that sampling error in DOD likely reduced our ability to measure spatiotemporal patterns in *Bd* spread in the Venezuelan region (Wave 2, see error analyses below).

### Rates of Spread

*Bd* can spread rapidly, moving across entire countries in less than 5 y and across northern South America in approximately 20 y. The estimated rates of spread of *Bd* in the Andes are generally consistent with rates reported from Central America [6], viz., 25–282 km/y (Figure 3 and Table 1). The upper range of these rate estimations (202 and 282 km/y) results from the record of *Bd* in Peru (A. tricolor, 1998) and a 1-y difference between A. eusbianus and A. quimbaya approximately 280 km apart.

### Incorporating Error into Estimates of LYO and DOD

**Figure 5:**
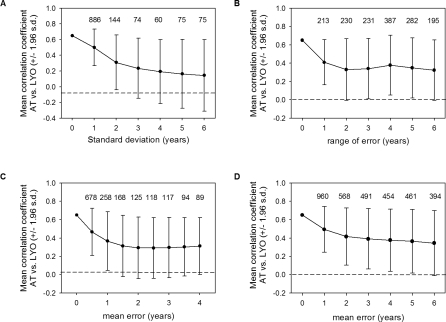
The Strength of the Relationship between AT and LYO Decreased as Error Was Added Systematically to LYO Plotted are the mean correlation coefficients (± 95% CI) for the relationship between AT and LYO. Numbers above the points represent the number of simulated runs that found a relationship equal to or greater in magnitude than that found in [17]. (A) Error is added symmetrically around LYO from a normal distribution of mean zero and increasing standard deviation. (B) Error is added forward in time from a uniform distribution. (C) Error is added forward in time with respect to LYO and is sampled from a Poisson distribution. (D) Error is added forward in time from an exponential distribution.

The previously published [17] relationship between AT and LYO was not robust to the incorporation of sampling uncertainty in LYO data (Figure 5). The original analysis [17] suggested a correlation coefficient of 0.65, relating LYO and AT from the previous year. However, using a normal error distribution of mean zero, the correlation coefficient quickly fell to 0.14 after the standard deviation of LYO was increased to 6 y. Even when the standard deviation was only 2 y, the 95% confidence interval overlapped zero, and only 1.4% of the simulated trials resulted in a correlation coefficient equal to or greater in magnitude to that of the original analysis. The decrease in the magnitude of correlation coefficients was not as great using Poisson, exponential, or uniform error forward in time, but suggested an overall poor relationship. Using a Poisson error distribution, the correlation coefficient dropped to an average of 0.30 for error ranging from 1.5 to 5 y. Further, no more than 2.6% of simulated correlation coefficients showed a value equal to or greater than the original result after 1.5 y, and the confidence interval of the result overlapped zero. After 2 y of error sampled from a uniform distribution, the correlation coefficient averaged 0.34 in simulated results, and only 2.6% of the simulated correlation coefficients were greater than or equal to the original result when including any amount of error. Similarly, after 2 y of error sampled from an exponential distribution, the correlation coefficient averaged 0.42 in simulated results, and only 5.7% of the simulated correlation coefficients were greater than or equal to the original result when including any amount of error. Interestingly, all of the correlation coefficients remained positive when error was added. This positive relationship is likely the result of both *Bd* spread and AT being positively correlated with time.

**Figure 6:**
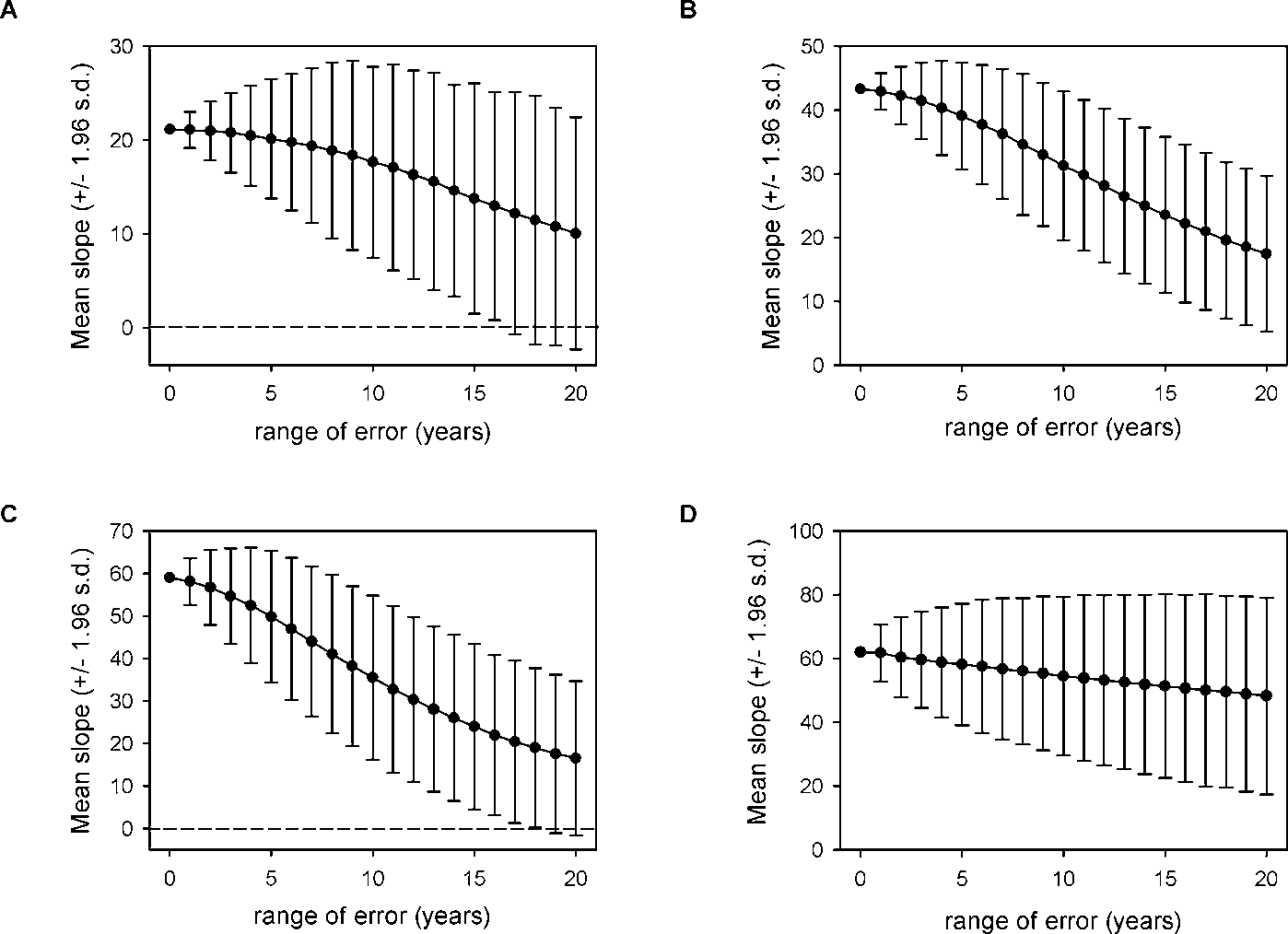
The Relationship between Time since the Earliest DOD (a Proxy for the Introduction of *Bd*) and Distance of Spread of *Bd* Was Robust to the Addition of Sampling Error (A) When adding error backward in time from a uniform distribution, the relationship in Wave 1 remained statistically significant when random error up to 16 y was applied, in (B) Wave 3a up to 20 (+) y, in (C) Wave 3b up to 18 y, and in (D) Wave 4 up to 20 (+) y was applied.

Unlike the relationship between AT and LYO, the relationship between time since the earliest DOD and distance of spread of *Bd* was robust to additional sampling error (Figure 6). When adding error backward in time from a uniform distribution, the relationship in Wave 1 remained statistically significant when random error up to 16 y was applied, in Wave 3a and Wave 4 up to 20 (+) y, and in Wave 3b up to 18 y.

### Elevational Patterns of Extinction

**Figure 7:**
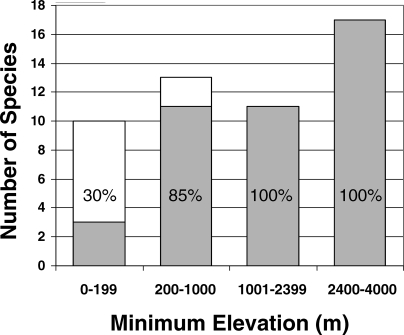
Percentage of *Atelopus* Species Declined or Extinct by Elevation in our Study Area Bars show the number of species at each elevation category while gray depicts the number of species in decline and white depicts stable species. The percentage of species in decline is written on each bar. Total number of species included in the analysis was 51.

An increasing percentage of *Atelopus* species declined as elevation increased (chi-square = 13.16, *df* = 2, *p* = 0.0014), with 100% of species occurring above 1,000 m having declined prior to 2004, while only 30% of those species near sea-level declined (Figure 7).

### Histology

None of the 64 individual frogs (Table S1) examined histologically that were collected from Monteverde prior to the documented declines in 1987 were infected with *Bd* (*p* = 0, 95% confidence interval [CI] = 0%–5.6%). Although not conclusive, this suggests that *Bd* was not endemic in frogs at the site prior to that time.

Our statistical analyses of the histological results of Ecuadorian frogs [57] support our hypotheses that it was unlikely (1) that *Bd* was present in Ecuadorian amphibians prior to 1980 (*N* = 32, all negative, 95% confidence limits [CL] = 0–0.108881), or (2) that *Bd* was present and infecting Atelopus ignescens in Ecuador prior to this species' decline in 1988 (*N* = 89, all negative, 95% CL = 0–0.040601).

## Discussion

Our results make two main points. First, we present analyses supporting a classical pattern of disease spread across naive populations, at odds with the CLEH proposed by [17]. Second, our analyses and re-analyses cast doubt on CLEH. We discuss these findings in more detail below.

### Evidence for a Spreading Pathogen

Our analyses of data for declines of amphibians in the Andean region of South America identified spatiotemporal patterns that were robust to sampling error in DOD and consistent with the spread of an introduced, invasive pathogen [23–26], as has been documented for *Bd* in Central America ([6] and additional results presented here). The oldest records of *Bd* in South America are 1980 in Ecuador [12], 1981 in Brazil [16], and 1986 in Venezuela [53], although the earliest reported declines of amphibian populations are in the “mid-1970s” in Venezuela [53], mid-1980s in Ecuador [12], and 1979 in Brazil [59]. Targeted searches for *Bd* in museum specimens collected from these areas at these times may produce older records.

Results of histology support our hypothesis that *Bd* was absent from Ecuador prior to 1980 and was introduced as an exotic pathogen. We use that date as the original introduction from which we modeled the spread along Waves 2, 3a, 3b, and 4. Within 7 y, *Bd* was found throughout Ecuador, supporting the hypothesis that it is an exotic pathogen invading naive populations.

Of particular importance when evaluating our results in light of the known errors in DOD are the results of the Monte Carlo simulations. The statistically significant spatiotemporal regressions of *Bd* dynamics were robust to even large amounts of error in DOD. These results strongly indicate that even if some of our DOD data were incorrect (which is likely given the quality of the *Atelopus* database), the wave-like pattern would remain robust.

Interestingly, the pattern of wave-like spread was more robust to sampling error than the CLEH. This result is likely due to resolution of LYO and DOD. Both LYO and DOD have inherent error because neither was collected with foreknowledge of pending declines—unlike data in Central America [6]. As such, the resolution of data is on the scale of roughly a decade (on average 11.2 y ± 8.2 SD). Because the CLEH predicts that declines occur *one* year following a relatively warm year [17], one should not expect the trend to hold up to the sampling error inherent in LYO or DOD. Indeed, LYO and DOD would need to have a resolution of 1 y or less to adequately evaluate the CLEH. The hypothesis of an epidemic wave, on the other hand, does not require such fine-scale resolution in time. There is no minimum distance that the disease needs to spread in a single year, just that the distance increases with an increasing number of years. In this case, data quality and the number of points in the relationship become especially important. This point can be demonstrated well with Wave 2. If A. sorianoi is excluded from Wave 2, the relationship for spread goes from not statistically significant (*p* = 0.32) to statistically significant (*p* = 0.0153; β = 17.2; *R* ^2^ = 0.92). In this case, Wave 2 would be nearly as well supported as Wave 1, but note that the slope of the relationship is rather low, so outliers will greatly affect this wave. Such a finding underscores the need for quality data.

In addition to identifying four regions with wave-like spread, results indicated that declines, extinctions, and epidemics occurred years after the leading edge of the wave had passed. This has likely prevented others from recognizing the invasion pattern, such as the logistic regressions using the entire dataset based on geographic coordinates performed by Pounds et al. [17]. Some of these events are likely real, and are typical of spreading pathogens, described as “great leaps forward” [60,61]. Other jumps likely are not real, but result from a lack of accurate field data, human facilitation of movement [61], effects of small-scale geography, effects of population dynamics, or from limitations of our large-scale approach. For example, each location represents the estimated geographic centroid of a species' distribution, not necessarily the precise location of *Bd* infection. We note that the Central American wave, measured with almost no error, had the strongest relationship between distance and time, and showed the least amount of variation in rates of spread despite being measured over the smallest spatial scale. Thus, the sampling error in the South America data may be large, and given the relatively rapid rate of *Bd* spread, makes smaller-scale detections of patterns, such as the spread of *Bd* through Ecuador, impossible to discern.

Geography may influence gene flow in plants and animals [62], including amphibians [63] and their pathogens [20]. In the case of chytridiomycosis, it is likely that certain habitats will promote the survival and spread of *Bd* (e.g., mountain chains and river valleys), other habitat features are likely to slow its spread (e.g., deserts and lowlands), and some are sufficiently remote or isolated such that invasions may be delayed (e.g., isolated highland ponds and upper reaches of minor tributaries). Finally, anthropogenic facilitation of *Bd* spread is also likely, perhaps along highways, seasonal routes of livestock herding, and other travel routes that might produce a pattern of nonlinear declines.

### Rates of Spread

Our estimates of *Bd* movement across all regions are quite crude, and we note that studies at small scales have found the slowest rates (e.g., 1.1 km/y in California; V. Vredenburg, personal communication), regional scales provide intermediate estimates (e.g., 26 km/y in Costa Rica and Panama [6]), and continental comparisons produce the highest rates of spread (282 km/y South America; this study). The scale of our study and, especially, the nature of the data available cannot provide robust estimates of fine-scale disease spread—either historical or predictive—for the region. However, these results can guide searches for historic records, and direct future research into finding evidence of multiple invasions or investigating potential mechanisms of transmission and spread. Ultimately, this suggests that different processes drive *Bd* spread at different scales. For example, rapid movement between and across continents may be caused by human-facilitated movements [64], whereas regional spread within continents is by natural dispersal through riparian corridors, and local spread via amphibian dispersal and transfer of *Bd* among individuals.

### Support for CLEH?

Our analyses and re-analyses of data related to the CLEH all fail to support that hypothesis. First, our simulations indicate that the correlations reported in [17] are not robust and fail with the inclusion of even small levels of error. This is especially important when considering the 1-y time lag between climate events and frog declines that is central to the CLEH model; that approach is very sensitive to slight errors in dates, and the effects of such error were not explicitly examined in that study. While acknowledging the uncertainties in the frog data [17], the potential effects of error in LYO were not explicitly tested at any level of analyses. We note that our simulations not only explored the effects of bias in estimates of LYO (i.e., forward sampling), but also unbiased random errors (errors from a uniform distribution). We consider our tests of error on LYO conservative in that our maximum levels of error (6 y), was smaller than the observed discrepancies between DOD and LYO.

Second, we show the elevational pattern in *Bd* prevalence [17], a cornerstone of CLEH, was not a real biological pattern but instead was caused by the combined use of LYO data and an arbitrary time cutoff of 1998. Reanalysis of these data using the more accurate Status information [14] shows an elevational pattern at odds with CLEH. Our results are essentially the same as those originally reported [14], in which there is an increasing percentage of *Atelopus* species declining as elevation increases, so that 100% of species occurring above 1,000 m declined prior to 2004. In contrast, the use of 1998 as a cutoff date [17] completely changed the results of elevational patterns of extinction in the *Atelopus* database, and incorrectly produced an artifactual “mid-elevation peak” in extinction that was then used to support the CLEH.

Third, it was suggested [17] that *Bd* might be an endemic saprophyte that emerges as a pathogen as a result of climatic anomalies. This was a key element of the CLEH hypothesis and the conceptual basis for much of the analysis. If true, *Bd* should be present, but nonpathogenic, in populations prior to declines. Although we cannot rule this out, currently no evidence supports this hypothesis, including our surveys for *Bd* in museum specimens collected at Monteverde, Costa Rica, and those collected from throughout Ecuador [57]. In the case of A. ignescens in Ecuador, a large number of individuals were examined with histology; another sample was available for additional species throughout Ecuador prior to the oldest record of 1980 [57]. These data indicated only a 4%–11% probability that *Bd* was present in these populations, but undetected, prior to known declines. For Monteverde, that probability was estimated at 5.6%, although we had to combine results from many species. Given differences in species susceptibility to *Bd*, this approach does not estimate true prevalence, although this approach produced robust estimates for a decline in Panama [6]. It is never possible to prove that *Bd* was absent from a population, but demonstrating that the upper 95% confidence limit for its prevalence is low [6] may be adequate.

### Invasive Pathogen

*Bd* is hypothesized to be an emerging infectious disease [7,11,22] that has been introduced at various times and sites around the globe; vectors for such geographic spread are assumed to be nonnative frogs such as Xenopus laevis or Lithobates catesbeianus [64,65], but conclusive evidence of historical introductions are unlikely to be identified. A more compelling indicator of multiple introduction events lies in the genetic signature of the pathogen itself, as described in fine-scaled studies in the Sierra Nevada of western North America [20]. The debate as to whether *Bd* is an exotic pathogen spreading through naive populations, or whether it is an endemic pathogen whose emergence is stimulated by climate change is strikingly similar to the discussion of the origin of Ebola Zaire (ZEBOV; [27]). In the case of ZEBOV, the addition of genetic data to spatial and temporal patterns of outbreaks strongly supported the hypothesis that ZEBOV had recently spread across the region. This approach [27] is necessary to understand the historical patterns of introduction and spread in South America, and at *Bd-*endemic sites in Lower Central America, where a single epidemic wave has been hypothesized [6].

Although we propose at least three independent introductions of *Bd* into South America (Ecuador, Venezuela, and Brazil), we note that our estimated rate of spread from *A. peruensis* to *A. tricolor*, in Peru, is high (∼202 km/y, suggesting perhaps that another introduction has occurred somewhere to the South. *Bd* has been present in neighboring Argentina since at least 2002 [66], but has yet to be reported from Chile [1]. However, one of the earliest enigmatic [2] declines in the Neotropics was that of Rhinoderma rufum, which was regularly seen in temperate forests and bogs in Chile until 1978 when it completely disappeared. Like the US and Europe, Chile received shipments of *Xenopus* from South Africa, and introduced populations were established there in the wild by 1944 [64]. We encourage surveys for *Bd* in preserved and living amphibians from Chile to test this hypothesis.

### Conclusions

Our analyses support a hypothesis that *Bd* is an introduced pathogen that spreads from its point of origin in a pattern typical of many emerging infectious diseases. Furthermore, although we acknowledge that climate change represents a serious threat to biodiversity, and likely influences endemic host–pathogen systems, the available data simply do not support the hypothesis that climate change has driven the spread of *Bd* in our study area.

That *Bd* is spreading into new populations and new regions is not surprising. What has been less clear is whether climate change has played a role in the emergence or spread of the disease. It has been mentioned [17] that patterns of decline could indicate simple wave-like spread of epidemic disease—apparently to reconcile the evidence of such in Central America [6]. The simple spatiotemporal pattern of *Bd* epidemics in Central America [6] has never been refuted, and has been supported by predictions by conservationists working in the region [28], and our analyses here corroborate a generalized pattern of spread in South America.

Disease dynamics are the result of a complex process involving multiple factors related to the hosts, the pathogen, and the environment that may affect disease patterns on many spatial scales. Disease dynamics are affected by micro- and macro-climatic variables [36], and such synergistic effects likely act on *Bd* and amphibians, because life histories of both the host and pathogen are directly influenced by both humidity and temperature. For example, environmental changes may cause some enzootic pathogens to become epizootic by causing them to expand their distribution into new areas, alter host behavior, or affect transmission rates [36,67]. Global climate change will directly influence amphibians but will also affect them indirectly through synergisms with habitat alteration, environmental contamination, diseases, and other challenges. A singular failure of recent studies involving climate and the amphibian crisis has been to seek evidence of interaction between temperature and disease across large areas that include sites where *Bd* is nonendemic, where it is endemic, and where it is epidemic, and to do so against a confounding backdrop of steadily increasing temperature regimes [17,18].

A straightforward example of how climate change might drive amphibian extinctions from invasion of *Bd* into new environments was described for the Peruvian Andes [15]. Glacial retreat produced new meltwater ponds that were colonized by three species of frogs [15] and later by *Bd*. This event was followed by die-offs of adults, and declines in metamorphic juveniles and tadpoles. Regional climate analyses [38] indicated this area of frozen ice and snow was not within the appropriate thermal envelope for *Bd*, but measurements of water temperature indicated that solar heating was capable of warming ponds to levels tolerable by *Bd*.

Future studies need to first determine whether or not *Bd* is present at the site, and then determine how long it has been there. Scientists must acknowledge that amphibians are proxies for the presence of *Bd* and carefully examine data related to declines to accurately portray the timing of infection. LYO data likely are inaccurate, and researchers should attempt to discover the time of *Bd* arrival or use DOD as more accurate estimates. Where *Bd* is endemic, complex synergistic interactions exist among the hosts, the pathogen, and climatic variables [68]. If *Bd* is already endemic (either naturally or introduced), then populations should be studied and monitored to evaluate relative susceptibility to fatality and catastrophic declines [69]. Where *Bd* is not yet present (e.g., currently Panama east of the Panama Canal), key challenges will revolve around preventing introductions and developing plans to conserve species- and genetic-level diversity in the event of epidemics (e.g, the Madagascar Amphibian Action Plan).

## Supporting Information

Table S1 List of Monteverde Specimens Examined for *Bd*
(32 KB DOC)

## Abbreviations

AT: air temperature
*Bd*: Batrachochytrium dendrobatidis
CLEH: climate-linked epidemic hypothesis
DOD: date of decline
GAA: Global Amphibian Assessment
LYO: last year observed
