# Supplementary material for: Riding the Wave: Reconciling the Roles of Disease and Climate Change in Amphibian Declines
Source: PLoS Biol. 2008 Mar 25;6(3):e72. doi: 10.1371/journal.pbio.0060072 (PMC2270328; doi:10.1371/journal.pbio.0060072)
Supplement: Table S1 — (32 KB DOC) [file pbio.0060072.st001.doc]

Table S1 1. List of Monteverde specimens examined for *Bd*.

| Species | Number |
| --- | --- |
| *Agalychnis annae* | 2 |
| *Atelopus varius* | 1 |
| *Bufo coniferus* | 1 |
| *Centrolene prosoblepon* | 8 |
| *Eleutherodactylus andi* | 5 |
| *Eleutherodactylus angelicus* | 13 |
| *Eleutherodactylus crassidigitus* | 6 |
| *Eleutherodactylus diastema* | 1 |
| *Eleutherodactylus fitzingeri* | 3 |
| *Eleutherodactylus melanostictus* | 8 |
| *Hyalinobatrachium colymbiphyllum* | 1 |
| *Hyalinobatrachium fleishmanni* | 1 |
| *Hyalinobatrachium valerioi* | 2 |
| *Hyla angustilineata* | 5 |
| *Hyla pseudopuma* | 1 |
| *Hyla rivularis* | 2 |
| *Phyllomedusa lemur* | 2 |
| unknown | 2 |
